# Genomic Polymorphism of Human Papillomavirus Type 52 in Women from Northeast China

**DOI:** 10.3390/ijms131114962

**Published:** 2012-11-15

**Authors:** Zhengrong Sun, Zhitao Lu, Jianhua Liu, Guili Wang, Weiqiang Zhou, Lianxia Yang, Chao Liu, Qiang Ruan

**Affiliations:** 1 Virus Laboratory, The Affiliated Shengjing Hospital, China Medical University, Heping District, Shenyang 110004, China; E-Mails: sunzr@sj-hospital.org (Z.S.); adlyn06040112@yahoo.com.cn (Z.L.); liujh@sj-hospital.org (J.L.); xiaolxiao@163.com (G.W.); zhgrw@126.com (W.Z.); yanglianxia870501@163.com (L.Y.); henryliu17@hotmail.com (C.L.); 2 Department of clinical laboratory, The Affiliated Shengjing Hospital, China Medical University, Heping District, Shenyang 110004, China

**Keywords:** HPV 52, *E6*, *E7*, *LCR*, *L1*, genomic polymorphism

## Abstract

Human papillomavirus (HPV) 52 is an oncogenic HPV type prevalent in Asia. The aim of the study was to analyze HPV 52 genetic variations in women from Northeast China. To explore the intratypic variants of HPV 52, the genomic regions of *L1*, *E6*, *E7* and long control region (*LCR*) of HPV 52, which have been identified in women from Northeast China by HPV GenoArray test, were analyzed. Twenty-five mutations were identified in the regions examined. Of the mutations found in the *L1* gene, three novel nonsynonymous mutations of *C5640T*, *A5641T* and *G5642A* were located within the region that encodes the binding domain of neutralizing antibodies against HPV 52. Although four variations were identified in HPV 52 *E6* and *E7* genes, no significant association was found between the mutations and the cytological lesion of the patients. Eight mutations, including a novel *CTT7681*–*7683* deletion, found in the *LCR* of HPV 52 encompassed the known transcription binding sites, which may possibly affect the transcription of the oncogenic genes of *E6* and *E7*. The most prevalent HPV 52 variant in women from northeastern China belongs to clade L1-LN-A. The genetic variations of HPV 52, including three novel nonsynonymous mutations of *C5640T*, *A5641T* and *G5642A* in the *L1* gene and a novel *CTT7681–7683* deletion in the LCR, were first documented in strains from women in Northeast China. The statistical result showed no associations between the variants and the severities of the infected women. These findings provide new data regarding gene variations of HPV 52.

## 1. Introduction

Oncogenic human papillomavirus (HPV) is a major cause of cervical cancer. HPV 16 and HPV 18 are highly prevalent in all regions of the world [1,2], followed by HPV 31, 33. Although rarely found in western countries, HPV 52 and 58 are prevalent in Asian populations, especially in China [3]. Accumulated studies have shown that HPV 52 and 58 are relatively more prevalent among HPV-positive women from Asia than in other places with only 11.5%–28% of prevalence across the full spectrum of cervical neoplasia [3–5]. It has been estimated that worldwide HPV prevalence is 10.4% in women with normal cervical cytology [6], but less than 1% of the infected women develop malignant lesions [7]. It is clear that persistent infection with specific high-risk HPV types is a strong marker for progressive CIN disease [8]. Recent studies have revealed that persistence of high-risk HPV infection might be associated with virus intratype variants. The HPV intratype variants are defined as having nucleotide sequence variations no more than 2% in the coding region and 5% in the noncoding regions of the viral genome with respect to the prototype [9]. Concerning HPV intratype variants, the most extensive studies have been conducted on HPV16 and HPV18 [10,11]. However, few data about HPV 52 intratype variants has been achieved so far.

Amino acid changes may affect the transforming activity of the E6 and E7 oncoproteins; those in the L1 protein may affect the efficacy of viral infection or alter viral antigenicity [12,13]. Mutations in the long control region (*LCR*) may affect virus replication rates and transcriptional activity of *E6* promoters [14,15]. In this study, the genetic variability of *LCR*, *E6*, *E7*, and *L1* genomic regions of HPV 52 was analyzed. Studies on HPV 52 variants in strains from cervical disease patients may improve the understanding of the molecular mechanisms underlying disease progression and transformation.

## 2. Results

### 2.1. Characteristics of the Study Populations and Distribution of HPV 52

For the HPV types found from the study population, 8.8% (72/815) were solely infected by HPV 52. Samples with multiple HPV positives were excluded to avoid confounding the results. Analyses of the *L1*, *E6*, *E7* and *LCR* genes were performed on all the single HPV 52 positive samples where sufficient materials were available. From the 72 samples, 60 sequences of *L1* gene, 66 sequences of *LCR*, *E6* and *E7* were obtained, respectively. Tables 1 and 2 show the sites of variations and changes in amino acid sequences in *L1*, *LCR*, *E6* and *E7* genes, respectively, compared with the HPV 52 prototype. The histologic diagnoses of the women whose infected strains were sequenced successfully for the *L1* gene were as follows: 11 normal cases (18.3%), 30 cervicitis (50.0%), 6 Ascus (10.0%), 3 CIN 1 (5%), 8 CIN 2 to 3 (13.3%) and 2 invasive cervical cancer (ICC) (3.33%) (Table 1); those for the *LCR*, *E6* and *E7* genes were as follows: 12 normal cases (18.2%), 34 Cervicitis (51.5%), 7 ASCUS (10.6%), 3 CIN 1 (4.55%), 8 CIN 2 to 3 (12.2%) and 2 ICC (3.03%) (Table 2).

### 2.2. Analysis of HPV 52 Genetic Variability

Sequence variations observed in HPV 52 clinical strains are summarized in Tables 1 and 2. Compared with the HPV 52 reference sequence (No.NC_001592), two strains were “L1-prototype-like,” and the remaining 58 strains were grouped into three different variants and named according to their frequency of variations as L1-LN-A, B and C (Table 1). The most prevalent HPV 52 variant in women from northeast China belongs to clade L1-LN-A (81.7%, 49/60). The statistical analysis by binary logistic regression showed that the variants of L1-LN-A, L1-LN-B and L1-LN-C were not associated with the severities of the infected women with the odds ratio (OR) of 0.444 (95% CI 0.094–2.093, *p* = 0.305), 1.741 (95% CI 0.162–18.675, *p* = 0.647) and 1.278 (95% CI 0.127–12.806, *p* = 0.835), respectively (Table 1).

Based on their variation rates among the four analyzed genes, the proportion of polymorphic nucleotides was significantly greater in the *LCR* than in that the other three genes: eight variation sites over 878 nt (0.91%) in HPV 52 *LCR* variants, compared with two variation sites (0.45%) over 447 nt in E6 variants, two variation sites (0.67%) over 300 nt in *E7* variants and thirteen variation sites (0.82%) over 1590 nt in *L1* variants. Deletions were found only in *LCR*. Based on the variations of *E6*, *E7* genes and *LCR*, the strains were clarified into four groups (Table 2). The statistical analysis by binary logistic regression showed no association between the first two groups and the severities of the infected women with the odds ratio (OR) of 1.626(95% CI 0.380–6.957, *p* = 0.512) and 0.714 (95% CI 0.166–3.066, *p* = 0.651), respectively.

### 2.3. HPV 52 L1 Sequence Variations

Analyses of the complete sequences of *L1* gene revealed 13 different nucleotide mutations. Except for *C6917A*, all the other mutations in HPV 52 *L1* gene were not reported previously. Among them, three (23.1%) novel mutations of *C5640T*, *A5641T* and *G5642A*, which led to the Q26L amino acid change, were identified in nine strains (Table 1). The Q26L amino acid mutation was located in the strand β-B1 of L1 protein. All the others were synonymous mutations, including *A5571G*, *T5972C*, *G6111A*, *G6218A*, *T6701G*, *T6764G*, *A6794G*, *C6824T*, *C6917A* and *G7802A*. Furthermore, two nonsynonymous mutations of *A5641T* and *G5642A* were covariations. The localizations of HPV 52 L1 protein mutations are reported in Table 1.

### 2.4. HPV 52 E6 and E7 Sequence Variations

The complete *E6* and *E7* open reading frames were analyzed in strains from 66 patients. Compared to the reference sequence, all the obtained sequences harbored the nucleotide variation of *G350A* and 65 had the nonsynonymous mutation of *A379G* (K93R) in the *E6* gene (Table 2). The *A379G* (K93R) mutation was located in the strand H1 and the third predicted zinc finger of E6 protein. Analysis of the complete E*7* open reading frame showed two synonymous substitutions of *C751T* and *A801G*.

### 2.5. HPV 52 LCR Sequence Variations

Compared to the reference sequence, *LCR* sequences had eight nucleotide mutations (Table 2). Among them, the *CTT 7681*–*7683* deletion was a novel mutation found in all of the studied strains. Besides the *CTT 7681*–*7683* deletion, another three mutations of *G7622A*, *T7624G/C* and *G7861A* were present in all obtained sequences, too. The other frequent polymorphic sites included *A7657C*, *T7659C*, *G7712C* and *A7865G*, that were detected in more than 64 strains. One covariation pattern and a statistically significant association were found among *A7657C*, *T7659C*, *G7712C* and *A7865G* mutations. (*p* = 0.01, *phy* = 1 for the associations).

All of the eight point mutations identified in *LCR* encompassed, and thus potentially affected, the proposed binding sites for transcription factors. In particular, the nucleotide substitutions of *G7622A* and *T7624G/C* were located in the TATA binding site, as well as *C/EBP* and *SRY* binding sites. The *CTT7681–7683* deletion was located in the *NF-E2* and *AP-1* binding sites; the nucleotide substitution of *G7861A* was located in the *Oct-1* binding site. The variations of *A7657C*, *T7659C*, *G7712C* and *A7865G*, which were predicted at the binding sites for *AP-1*, *HSF*, *MAT alp*, *Skn-1* and *HFS*, introduced additional putative binding sites for cellular proteins, such as Cap, Cdxa, Skn-1, and Oct-1 or HSF. Putative binding sites for cellular proteins SRY, and others, were also affected by less frequently encountered variations (data not shown). However, the binding sites for the HPV E2 protein were conserved in all the strains.

## 3. Discussion

Globally, the most prevalent HPV type is HPV-16, detected in approximately 40% of high-grade cervical lesions [17], and followed by HPV 18. The two viruses are responsible for about 70.1% of cervical cancers in the world. Therefore, research efforts have focused mainly on these two viral types and a prophylactic vaccine is available currently for prevention of HPV 16 and 18 infections [18,19].

However, the prevalence of other high-risk HPV types varies among different countries and even throughout regions of the same country [20]. In recent years, it has been reported that HPV 52 ranks fourth in cervical cancer cases within some Asian counties [17], and the fourth most prevalent types in patients with high-grade cervical lesions in Northern China [5].

Researches on genetic variability of HPV variants may increase the understanding of the molecular mechanisms underlying disease progression and transformation. Indeed, a number of studies suggest that variants of the same HPV type are biologically distinct and may confer differential pathogenic risks [21]. In this study, the genetic variability of *LCR*, *L1*, *E6* and *E7* genes of HPV 52 was analyzed. Compared to the published data [22–26], several novel mutations were discovered either in the *LCR* and the coding regions of HPV type 52.

In a recent study, an increase prevalence of CIN3 was reported to associate with HPV 52 lineage C [26]. Results of another study showed that all cases of CIN3 or worse were associated with a combined group of lineages A and B and C, but not with the group of lineage D, resulting in an odds ratio (OR) estimate of infinity [27]. Furthermore, a study found that the nonprototypic LCR variant was associated with the persistence of HPV 52 infection compared to the prototype [28]. In our study populations, HPV 52 showed four main variants inferred on L1 sequences. The most prevalent HPV 52 variant was L1-LN-A. However, no association between the variants and the grade of cervical lesion of infected patients was found. Whether or not the different findings are the result of the variants found in different geographical areas still needs to be studied.

Except for C6917A, all the other mutations in the HPV 52 *L1* gene were not described elsewhere [25]. The novel mutations of *C5640T*, *A5641T* and *G5642A*, which lead to amino acid change of Q26L, were detected in nine strains from patients with HSIL. The Q26L mutation was located in the strand β-B1 of the L1 protein, which may suggest that the variants were established to escape neutralization. This mutation may influence the folding of the L1 protein, with possible consequences on the immunogenicity of neutralizing epitopes, thus favoring persistence of the infection by viral evasion from neutralizing antibody responses [12,29].

HPV *E6* and *E7* genes are believed to be the main oncoproteins. In this study, the *E6* and *E7* gene sequences were relatively conserved, for only four mutations were identified in most of the studied HPV 52 strains, and no significant trends accordant with severity of cervical neoplasia were observed. Among the four mutations, only *A379G* (K93R) was nonsynonymous. The mutation *G350A* detected in the *E6* gene has been reported as *G350T* in the study of Ding *et al.*[30]. In accordance with Xin’s report [22], the unique *A379G* (K93R) variation was located in the strand H1 and the third predicted zinc finger of E6 protein and occurred in almost all of the HPV 52 positive samples.

Analysis of HPV 52 *LCR* revealed one covariation pattern. Notably, the novel *CTT7681*–*7683* deletion is firstly reported being detected in all the studied strains in Northern China. The biological meaning and geographical areas of such finding remains to be established. In addition, some of the mutations identified in *LCR* encompassed the proposed binding sites for *YY1* and *SRY* transcription factors. It is suspected that these mutations may affect the transcription of the downstream *E6* gene, though it remains to be determined by *in vitro* studies. Among HPV 52 isolates, the *LCR T7624G/C* substitution was detected in all of the samples tested. Interestingly, this mutation was suggested to be associated with the persistence of HPV 52 infection by Aho *et al*. [28].

The genetic variations of HPV 52 were first documented in this report in Northeast China. Additionally, the frequency distributions of HPV 52 variants in Northeast China were different from those reported in European and American populations.

## 4. Materials and Method

### 4.1. Study Populations

Cervical swabs of the studied populations were collected from patients who referred to the Department of Obstetrics and Gynecology, Shengjing Hospital of China Medical University for routine gynecological detections. The median age of the studied populations was 38 years (range, 20–66 years) at the time, the cervical scrapings were obtained. After giving informed consents, a total of 815 patients without a history of hysterectomy received an examination with the papanicolaou (Pap) smear, which collected cervical cells using ViraPap kits (Digene Diagnostic, Silver Spring, MD, USA) at study entry. Pap smears were graded according to the Bethesda system, which categorizes cytology grades into normal, atypical squamous cells of undetermined significance (ASCUS), low grade squamous intraepithelial lesion (LSIL), high grade cervical squamous intraepithelial lesion (HSIL) and cancer. Patients with LSIL or worse lesions were referred for a biopsy and treatment. Histological data were reviewed by expert pathologists to confirm the disease outcomes. Subjects gave a signed informed consent. The study protocol was approved by institutional ethical and research review boards of the participating institutions in the northeast of China.

### 4.2. HPV DNA Detection and Typing

Viral types were detected using the HPV GenoArray test kit (Hybribio Ltd, Hong Kong) according to the manufacturer’s instructions as described in the previous study [5]. In this method, flow-through hybridization was used based on the principle of Reverse Dot Blot Assay which was described in previous studies [16]. The method could classify 21 HPV subtypes including 12 established high-risk (HR) types (HPV-16, −18, −31, −33, −35, −39, −45, −51, −52, −56, −58, −59), three probable HR types (HPV-53, −66, −68) and six established low-risk (LR) types (HPV-6, −11, −42, −43, −44, and CP8304).

### 4.3. PCR Amplification

Samples positive with HPV 52 were further amplified with primer sets specifically designed for the regions containing the partial *LCR* fragment and complete *E6*, *E7* and *L1* ORF, respectively. PCR reactions were done in a 50 μL reaction volume containing 1× PCR buffer, 200 μM of each dNTP, 2 mM MgCl_2_, 20 pmol of each primer and 1 unit of Taq DNA polymerase (Takara, Japan). PCR amplicons were separated on 2% agarose gels and visualized by ethidium bromide staining under UV transillumination. In the case of no observed band on the gel, 2 μL PCR products obtained with outer primer pairs were used as a template for amplification with inner primer pairs. The primer sets and PCR amplification profiles are shown in the supplementary Table S1. A reaction mixture without template DNA was included as a negative control in every set of PCR.

### 4.4. HPV DNA Sequence Analysis

PCR amplicons were purified and applied to enzymatic extension reactions for DNA sequencing using the ABI PRISM Big-Dye Terminator Cycle Sequencing Ready Reaction Kit (Applied Biosystems, Bedford, MA, USA). Both strands of the recovered DNA were sequenced with the same forward and reverse primers as those used for PCR amplification. The sequencing reactions were run on an ABI 3730 XL DNA Analyzer (Applied Biosystems). The obtained HPV 52 sequences were aligned with the reference sequence (Genbank Accession No.NC_001592) on NCBI [31]. The multiple alignments were further refined by manual intervention.

### 4.5. Analysis of Transcription Factors Binding Sites in *LCR* Region

Potential binding sites for cellular and viral transcriptional factors within the HPV 52 *LCR* regions were searched by the TFSEARCH software, including sites for *AP-1*, *E2*, *GRE*, *NF-1*, *Oct-1*, *TATA*, *YY1*, *C/EBP*, *Sp1*, *SRY*, *AML-1a* and *c-Myc/c-Max*. Cut-off values and coincidence levels between consensus binding sites and the *LCR* sequence of HPV 52 type were adjusted in order to minimize both the number of negative and positive faults [32].

### 4.6. Statistical Analysis

Statistical analysis was performed using the SPSS software (version 17.0) [33]. The magnitude of the associations between HPV variants and HSILs of patients was assessed by binary logistic regression with odds ratios (ORs) and respective 95% confidence intervals (CIs). For examining distributions of HPV 52 variations with respect to disease severity, spearman correlation was employed. Two-sided, *p* < 0.05 was considered to be statistically significant.

## 5. Conclusions

In summary, the distribution of HPV 52 variants was investigated in a cohort of Chinese women. Some new variations were found among the studied strains. Although high ratios of HPV 52 *E6*, *E7*, *L1* genes and *LCR* variants were found in our strains, no variant was found to have a significant association with the severity of cervical lesions of infected women. Because the number of women with CIN and more severe lesions was limited in this study, whether certain variants could increase the severity of cervical neoplasia still needs to be confirmed by future studies with a larger number of cases.

## Supplementary Information



## Figures and Tables

**Table 1:** Variations of HPV 52 L1 gene in strains from patients with different grades of cervical lesions.

Categories	Variation of HPV 52 L1 at nucleotide position (1590 bp)													Grade of related cervical lesion							HSIL+ *vs.* Normal	
		
	^N^5	^N^5	^N^5	^N^5	^N^5	^N^6	^N^6	^N^6	^N^6	^N^6	^N^6	6	^N^7	Normal	Cervicitis	ASCUS	CIN-1	CIN2-3	CC	*N* (60)	OR (95% CI)	*p*
	6	6	6	5	9	1	2	7	7	7	8	9	8									
	4	4	4	7	7	1	1	0	6	9	2	1	0									
	0	1	2	1	2	1	8	1	4	4	4	7	2									
Reference nt	C	A	G	A	T	G	G	T	T	A	C	C	G	-	-	-	-	-	-	-	-	-
Reference aa	Q	Q	Q	-	-	-	-	-	-	-	-	-	-	-	-	-	-	-	-	-	-	-
aa position	26	26	26	-	-	-	-	-	-	-	-	-	-		-	-	-	-	-	-	-	-
aa mutations	L	L	L	-	-	-	-	-	-	-	-	-	-	-	-	-	-	-	-	-	-	-
L1-LN-A	-	-	-	G	C	A	A	G	C	G	T	A	A	10	25	4	3	5	2	49	0.444 (0.094 2.093)	0.305
L1-LN-B	T	T	A	G	C	A	A	G	C	G	T	A	A	0	2	1	0	1	0	4	1.741 (0.162 18.675)	0.647
L1-LN-C	-	T	A	G	C	A	A	G	C	G	T	A	A	1	2	1	0	1	0	5	1.278 (0.127 12.806)	0.835
L1-prototype-like	-	-	-	-	-	-	-	-	-	-	-	-	-	0	1	0	0	1	0	2	-	-

Note: Statistical analysis was calculated by binary logistic regression. A two-sided *p* < 0.05 was considered to be statistically significant. HPV 52 prototype (Genbank Accession No.NC_001592) was used as the reference. Nucleotide position in *L1* is reported at the top of the table according to the reference sequence. Amino acid substitutions are indicated under the corresponding amino acidic position. Nucleotide changes are shown by the corresponding letters. Dashes indicate positions at which no variation was found. Novel variations are labeled as “^N^”. HSILs, high grade cervical squamous intraepithelial lesion, including patients with CIN2, 3 and cervical cancer.

**Table 2:** Variability of HPV 52 E6, E7 and LCR gene regions in strains from infected patients. Nt positions

Nt positions	*E6*		*E7*		*LCR*									Grade of related cervical lesion							HSIL+ *vs.* Normal	
				
	-	-	-	-	7	7	7	7	^N^7	7	7	7	7	Normal	Cervicitis	ASCUS	CIN-1	CIN2-3	CC	n (66)	OR(95%CI)	*p*
	3	3	7	8	6	6	6	6	6	6	7	8	8									
	5	7	5	0	2	2	5	5	8	8	1	6	6									
	0	9	1	1	2	4	7	9	1	3	2	1	5									
Reference nt	G	A	C	A	G	T	A	T	CTT		G	G	A	-	-	-	-	-	-	-	-	-
Reference aa	A	K	L	Q	-	-	-	-	-		-	-	-	-	-	-	-	-	-	-	-	-
aa position	83	93	67	83	-	-	-	-	-		-	-	-	-	-	-	-	-	-	-	-	-
aa mutations	A	R	L	Q	-	-	-	-	-		-	-	-	-	-	-	-	-	-	-	-	-
																						
	A	G	T	G	A	G	C	C	delete		C	A	G	8	18	5	2	6	1	40	1.626 (0.380 6.957)	0.512
																						
Nucleotide mutations	A	G	-	G	A	G	C	C	delete		C	A	G	4	14	2	1	2	1	24	0.714 (0.166 3.066)	0.651
	A	G	T	-	A	G	C	C	delete		-	A	G	0	1	0	0	0	0	1	-	-
	A	-	T	G	A	C	-	-	delete		-	A	-	0	1	0	0	0	0	1	-	-

Note: Statistical analysis was calculated by binary logistic regression. A two-sided *p* < 0.05 was considered to be statistically significant. The HPV 52 prototype (Genbank Accession No.NC_001592) was used as the reference strain. Nucleotide position in *E6* is reported at the top of the table according to the reference sequence. Amino acid substitutions are indicated under the corresponding amino acidic positions. Nucleotide changes are shown by the corresponding letters. Dashes indicate positions at which no variation was found. Novel variations are labeled as “^N^”. HSILs, high grade cervical squamous intraepithelial lesion, including patients with CIN2, 3 and cervical cancer.
