# Supplementary material for: Genomic Polymorphism of Human Papillomavirus Type 52 in Women from Northeast China
Source: Int J Mol Sci. 2012 Nov 15;13(11):14962–72. doi: 10.3390/ijms131114962 (PMC3509622; doi:10.3390/ijms131114962)
Supplement: Supplementary file 1 [file ijms-13-14962-s001.pdf]

Supplementary Information

Table S1. The primer for HPV 52 E6, E7, LCR and L1 sequences

Name	Primer sequence	Position	Annealing temperature (°C)
E6/E7 F	CTTATACTAGTAAAAAATAGGG	12–33	56
E6/E7R	TTGTTTTTCTATTATTGCCTCTA	922–945	57
E6/E7-2F	GGGAAAACATTAGAAGAG	353–371	52
E6/E7-2R	ACGTTACACTTGGGTCACA	533–551	55
LCR F	TTGCACCCACATGAGTAACA	7414–7433	54
LCR R	AGTGCACACCTGGTGAGTAA	7891–7910	55
L1F	AGGTCCTGACATTCCCATTA	5504–5522	53
L1R	CATGACACAGACAATTACGCAACA	7191–7214	55
L1-1-641F	GTCCTCCCCTACAACTCATTA	6233–6253	57
L1-1-764R	AATCTGGATACTTACATACACTGC	6356–6379	56
L1-2-1123F	TTATGTGCTGAGGTGAAAA	6715–6733	53
L1-2-1327R	CTGTATGTGTCCTCCAAAGA	6919–6938	54

© 2012 by the authors; licensee MDPI, Basel, Switzerland. This article is an open access article distributed under the terms and conditions of the Creative Commons Attribution license (http://creativecommons.org/licenses/by/3.0/).
